# Supplementary material for: The Role of Treatment-Related Parameters and Brain Morphology in the Lesion Volume of Magnetic-Resonance-Guided Focused Ultrasound Thalamotomy in Patients with Tremor-Dominant Neurological Conditions
Source: Bioengineering (Basel). 2024 Apr 12;11(4):373. doi: 10.3390/bioengineering11040373 (PMC11047844; doi:10.3390/bioengineering11040373)
Supplement: Supplementary file 1 [file bioengineering-11-00373-s001.zip › bioengineering-2938360-supplementary.pdf]

Supplementary Materials

The Role of Treatment-Related Parameters and Brain Morphology in the Lesion Volume of Magnetic-Resonance-Guided Focused Ultrasound Thalamotomy in Patients with Tremor-Dominant Neurological Conditions

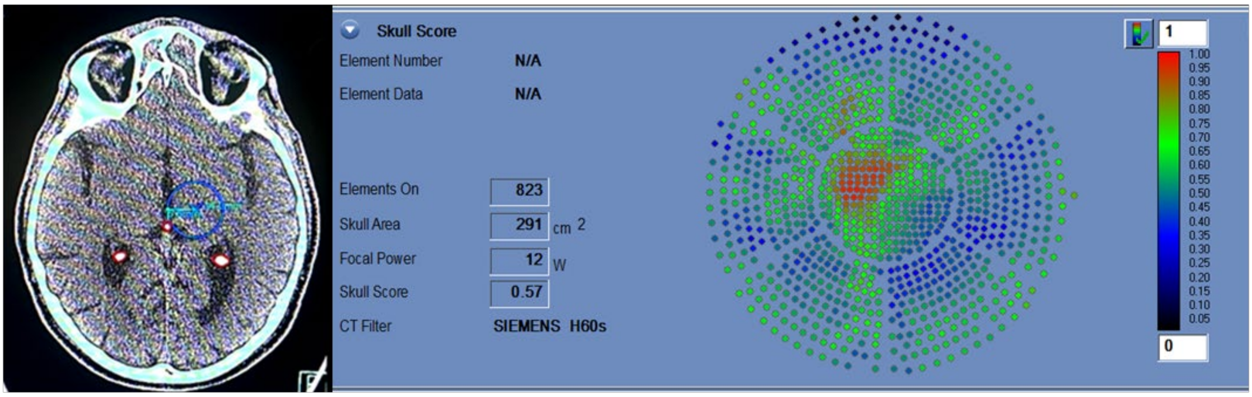

Figure S1. CT imaging. SDR, Skull Area and numbers of elements in use were calculated to evaluate treatment eligibility.

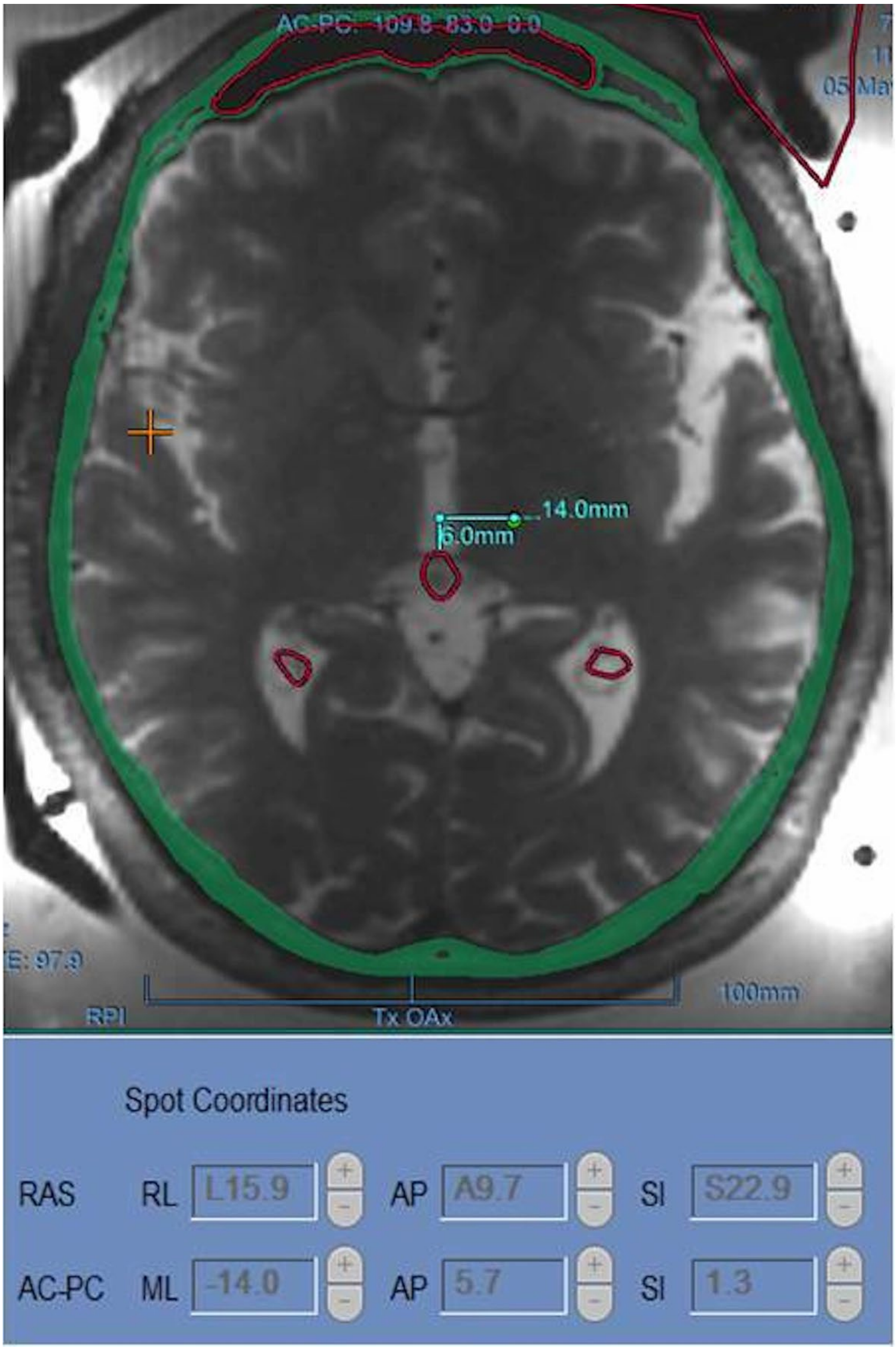

Figure S2. MRgFUS: Target position on the T2-weighted image.

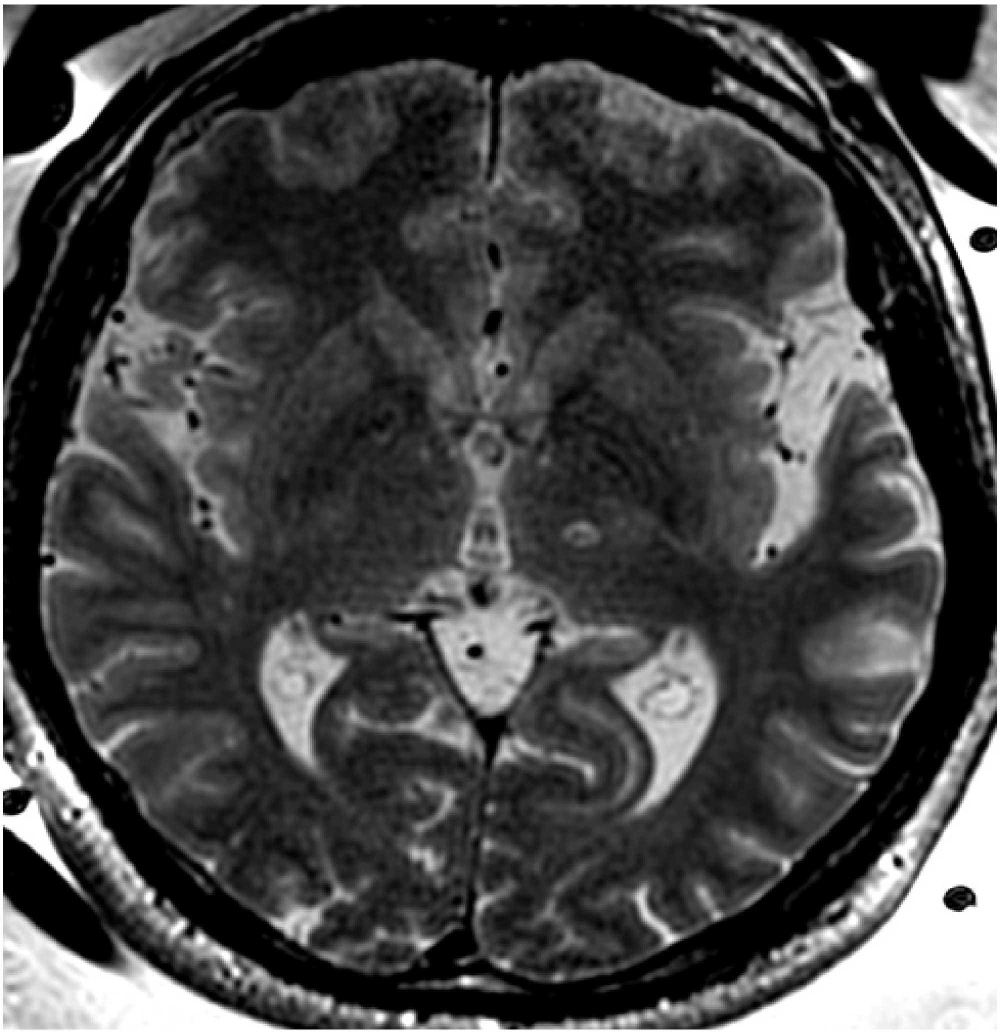

Figure S3. Axial FRFSE T2-weighted images after MRgFUS. The image shows the resulting thalamic lesion. The necrotic core appears hypointense and is surrounded by cytotoxic edema.

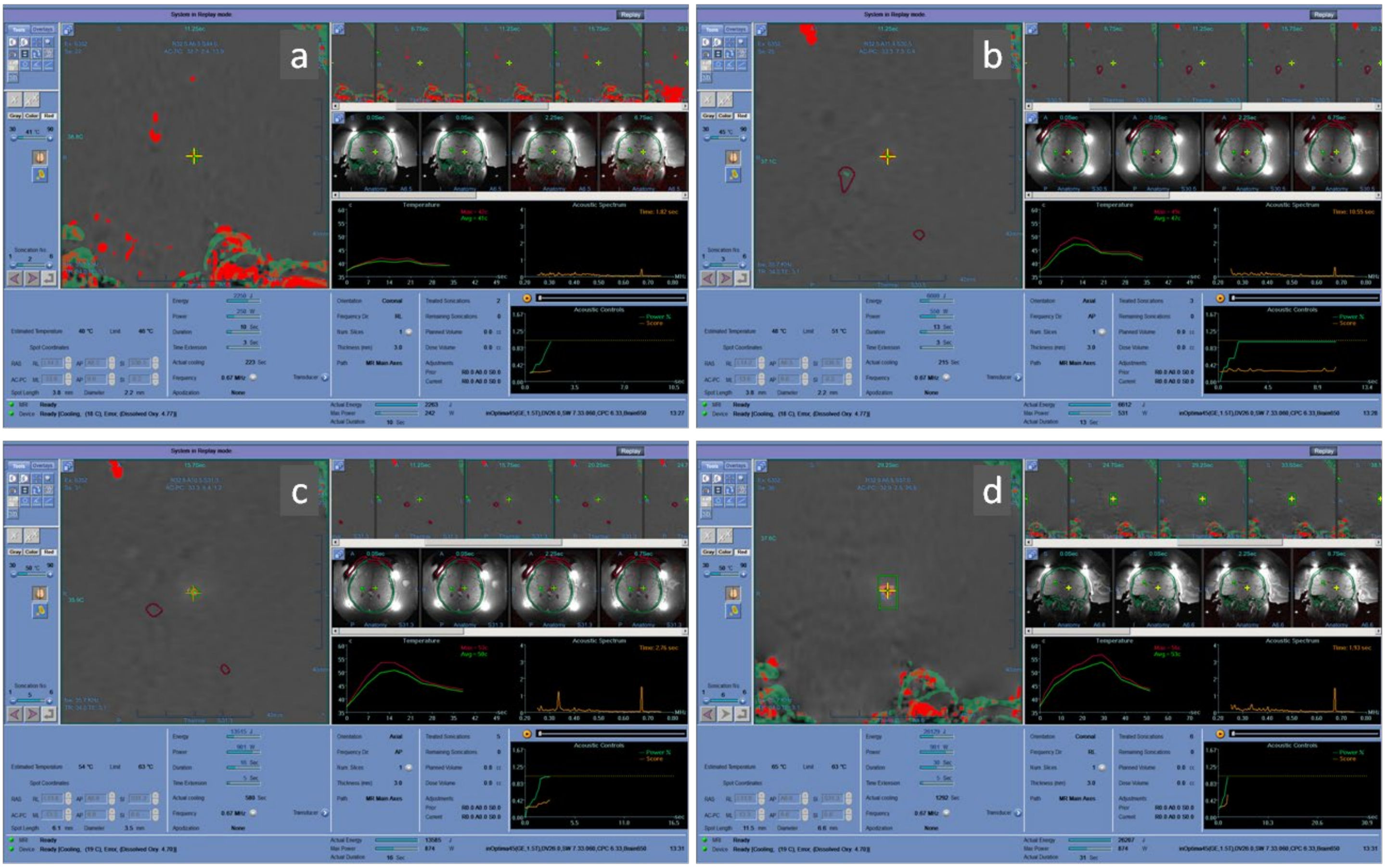

Figure S4. MRgFUS treatment- MR-thermometry procedure: a) Alignment; b) Verification; c-d) Treatment.

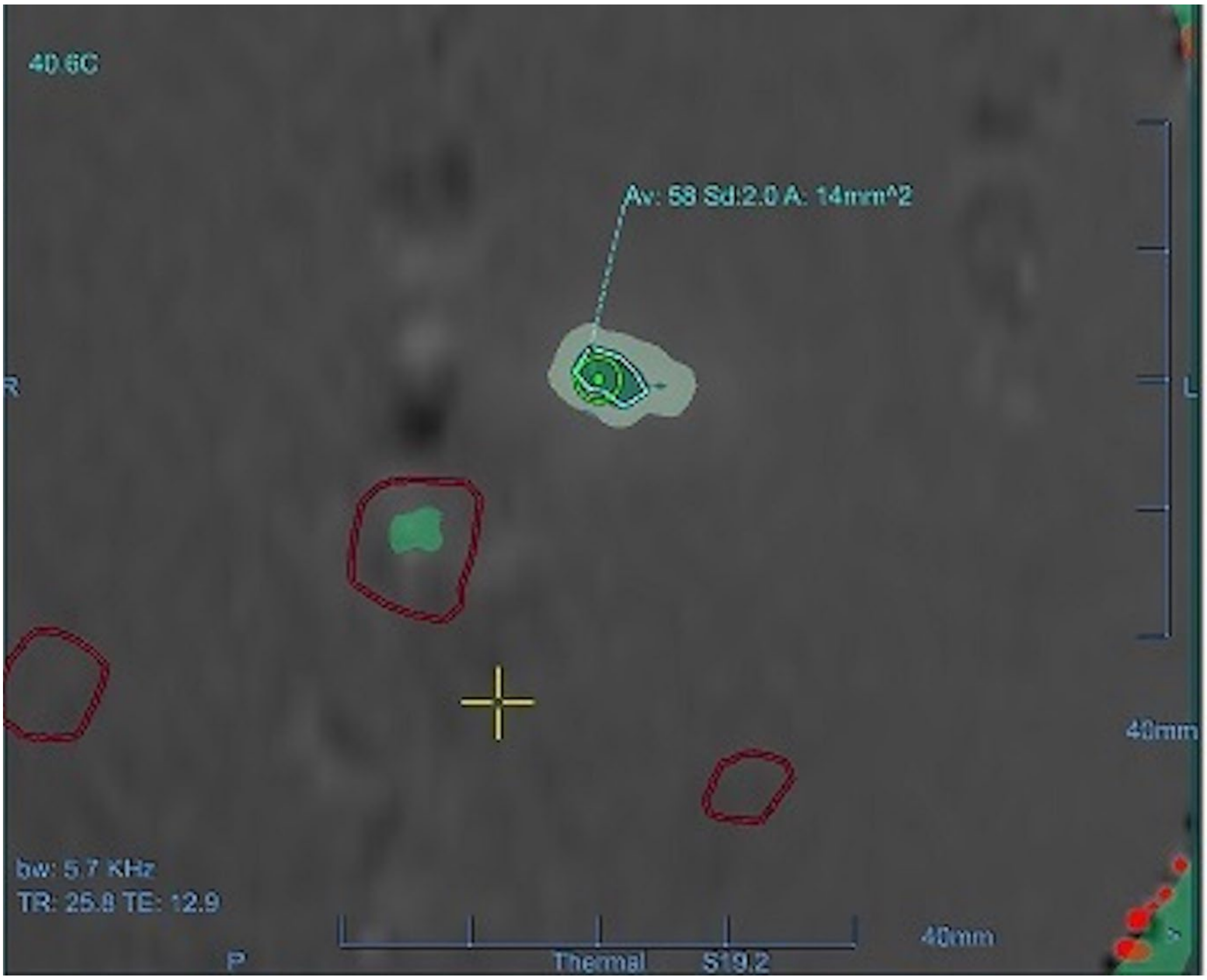

Figure S5. ATD is calculated from the thermometric map of the last sonication drawing a manual ROI on the target.

Table S1. Brain structural characteristics of the ET and PD population before MRgFUS treatment.

	**PD** (***n*** **= 19**)	**ET** (***n*** **= 17**)	***p-value*** [e]
Normalized GM Volume (cm³)	323.2 ± 34.2	329.7 ± 37.3	n.s.
Normalized WM Volume (cm³)	296.5 ± 37.1	288.4 ± 46.3	n.s.
Normalized CSF Volume (cm³)	378.5 ± 44.8	375.7 ± 52.8	n.s.
Normalized Brain Volume (GM+WM) (cm³)	619.9 ± 48.4	618.2 ± 49.3	n.s.
Total Cortical Thickness (mm)	2.51 ± 0.2	2.59 ± 0.1	n.s.

Abbreviations: n.s.: not significant

Table S2. Characterization of incident Adverse Events observed during MRgF.US

	PD (*n* = 19)	ET (*n* = 17)	*p-value* [e]
Motor Fluctuations	1	3	0.999
Dyskinesias	0	1	0.999
Impulse control disorder	1	2	0.999
Nausea	0	2	0.540
Leg edema	1	1	0.999
Hypotension	0	3	0.532
Daytime sleepiness	0	4	0.272
Anxiety/agitation	0	2	0.540
Skin reaction to rotigotine	0	2	0.540
Nausea/vomit	6	NA	NA
Scalp Numbness	1	NA	NA
Dizziness	6	NA	NA
Headache	2	NA	NA
Uncomfortable heat sensation	3	NA	NA
Anxiety	1	NA	NA
Neck pain	1	NA	NA

Abbreviations: AE, Adverse Event; MRI, Magnetic Resonance Imaging; PD-FUS, NA, Not Applicable.

Table S3. Clinical changes in motor and tremor scores before and after MRgFUS treatment.

	Baseline	Post-treatment	% Improvement	p-level
Total UPDRS score (PD patients)	37.3 ± 12.5	29.7 ± 11.7	28.8 ± 15.8	p < 0.001
Hemi-UPDRS *(affected side)* (PD patients)	23.3 ± 6.6	15.8 ± 7.3	34.7 ± 16.5	p < 0.001
Total CRST score (ET patients)	58.3 ± 15.2	36.4 ± 12.1	38.7 ± 11.8	p < 0.001
Hemi-CRST *(affected side)* (ET patients)	42.4 ± 12	20.3 ± 6.8	52.6 ± 13.9	p < 0.001
**% Clinical improvement for treated right-VIM ablation (n° 16)**				
Total UPDRS score	36.6 ± 11.7	29.6 ± 11.7	19.1 ± 10.1	p < 0.001
Hemi-UPDRS *(affected side)*	23.1 ± 7.3	16.1 ± 8.4	30.3 ± 8.1	p < 0.001
Total CRST score	51.2 ± 22.6	30.2± 16.8	41.1 ± 19.9	p < 0.001
Hemi-CRST *(affected side)*	35.5 ± 17.6	16 ± 10.95	54.9 ± 16.7	p < 0.001
**% Clinical improvement for treated Left-VIM ablation (n° 20)**				
Total UPDRS score	38.5 ± 14.7	30 ± 12.6	22.1 ± 11.8	p < 0.001
Hemi-UPDRS *(affected side)*	23.6 ± 5.8	15.1 ± 5.9	36 ± 5.9	p < 0.001
Total CRST score	60.4 ± 12.6	38.3 ± 10.4	36.6 ± 11.9	p < 0.001

Table S4. Simple linear regression among procedural MRgFUS parameters in all tremor dominant neurological patients (n°36).

	**Age**	**Disease duration**	**SDR**	**Skull area**	**n° of elements**	**n° of sonications**	**Max_Watt**	**Max_Joule**	**T°C_max**	**Max_Time**	**ATD**
**Age**		**n.s**	**n.s**	**n.s**	**n.s**	**n.s**	**n.s**	**n.s**	**n.s**	**n.s**	**n.s**
**Disease duration**			**n.s**	**n.s**	**n.s**	**n.s**	**n.s**	**n.s**	**n.s**	**n.s**	**n.s**
**SDR**				n.s	n.s	n.s	Rho = .-42; p = .01	Rho = -53; p = .0001	Rho = .44; p = .007	Rho = .-586; p= .001	n.s
**Skull area**					Rho = 530; p = .001	n.s	n.s	n.s	n.s	n.s	n.s
**n° of elements**						n.s	n.s	n.s	n.s	n.s	n.s
**n° of sonications**							n.s	n.s	n.s	n.s	n.s
**Max_Watt**								Rho = 72; p < .0001	n.s	Rho = 621; p < .0001	Rho = .-49; p = .002
**Max_Joule**									Rho = .-53; p < .001	Rho = .689; p < .0001	Rho = .-65; p < .0001
**T°C_max**										Rho = .-436; p = .01	Rho = 69; p < .0001
**Max_Time**											Rho = -56; p < .0001
**ATD**											

Skull Density Ratio (SDR), Max_Watt: maximum energy delivered (watt); Max_Joule: maximum power delivered (joule); Max_Time: maximum sonication time delivered; T°C_max: maximum mean temperature reached; ATD: accumulated thermic dose; N.S. not significant

Table S5. Simple linear regression among procedural MRgFUS parameters in PD patients (n°19).

	**SDR**	**Skull area**	**n° of elements**	**n° of sonications**	**Max_Watt**	**Max_Joule**	**T°C_max**	**Max_Time**	**ATD**	**Necrosis-volume**	**Edema volume**	**Delta Total UPDRS**	**Delta Hemi-UPDRS**
**SDR**		n.s	n.s	n.s	n.s	Rho = -.664; *p* = .002	n.s	Rho = -.597; *p* = .007	n.s	n.s	n.s	n.s	n.s
**Skull area**			Rho = .468; *p* = .043	n.s	n.s	n.s	n.s	n.s	n.s	n.s	n.s	Rho =- .469; *p* = .043	n.s
**n° of elements**				n.s	n.s	n.s	n.s	n.s	n.s	Rho = - .621; *p* = .005	Rho = - .464; *p* = .046	n.s	n.s
**n° of sonications**					n.s	Rho = .547; *p* = .015	n.s	n.s	n.s	n.s	n.s	n.s	n.s
**Max_Watt**						Rho =.729; *p* < .0001	Rho =-.558; *p* = .013	Rho = .611; *p* = .005	Rho = -.487; *p* = .035	n.s	n.s	n.s	n.s
**Max_Joule**							Rho = -.68; *p* = .001	Rho = .879; *p* < .0001	Rho = -.667; *p* = .002	n.s	n.s	n.s	n.s
**T°C_max**								Rho = -.612; *p* = .005	Rho =.715; *p* =.001	n.s	Rho = .477; *p* = .039	n.s	n.s
**Max_Time**									Rho = -.627; *p* = .004	n.s	n.s	n.s	n.s
**ATD**										n.s	n.s	n.s	n.s

	Necrosis volume	Edema volume	Delta_Total_UPDRS	Delta_Hemi_UPDRS
**Necrosis volume**		Rho = -.623; *p* =.004	n.s	n.s
**Edema volume**			n.s	n.s
**Delta_Total_UPDRS**				Rho = .956; *p* <.0001
**Delta_Hemi_UPDRS**				

Table S6. Simple linear regression among procedural MRgFUS parameters in ET patients (n°17).

	**SDR**	**Skull area**	**n° of elements**	**n° of sonications**	**Max_Watt**	**Max_Joule**	**T°C_max**	**Max_Time**	**ATD**	**Necrosis-volume**	**Edema volume**	**Delta Total CRST**	**Delta Hemi-CRST**
**SDR**		n.s	n.s	n.s	n.s	n.s	n.s	Rho = -.528; *p* = .029	Rho = .530; *p* = .029	n.s	n.s	n.s	n.s
**Skull area**			Rho = .642; *p* = .005	n.s	n.s	n.s	n.s	n.s	Rho = .548; *p* = .023	Rho = .495; *p* = .043	n.s	n.s	n.s
**n° of elements**				n.s	n.s	n.s	n.s	n.s	Rho = .502; *p* = .040	n.s	n.s	n.s	n.s
**n° of sonications**					n.s	n.s	n.s	n.s	n.s	n.s	n.s	n.s	n.s
**Max_Watt**						Rho =.674; *p* = .003	n.s	Rho = .515; *p* = .034	n.s	n.s	n.s	n.s	n.s
**Max_Joule**							n.s	Rho = .502; *p* =.040	Rho = -.640; *p* = .006	n.s	n.s	n.s	n.s
**T°C_max**								n.s	Rho =.657;	Rho =.672;	Rho = .589;	n.s	n.s

			p =.004	*p* =.003	*p* = .013		
**Max_Time**			Rho = -.488; *p* = *.047*	n.s	n.s	n.s	Rho = -.538; *p* = *.026*
**ATD**				n.s	n.s	n.s	n.s
**Necrosis volume**					n.s	n.s	Rho = -.545; *p* = *.024*
**Edema volume**						n.s	n.s
**Delta_Total_CRST**							Rho = .793; *p* <*.0001*
**Delta_Hemi_CRST**							
